# Glioblastoma Multiforme Oncogenomics and Signaling Pathways

**DOI:** 10.4137/cmo.s1008

**Published:** 2009-04-08

**Authors:** Okezie O. Kanu, Betsy Hughes, Chunhui Di, Ningjing Lin, Jinrong Fu, Darell D. Bigner, Hai Yan, Cory Adamson

**Affiliations:** 1 Lagos University Teaching Hospital, Lagos, Nigeria.; 2 Department of Oncology, Beijing Cancer Hospital, Peking University School of Oncology, Beijing, China.; 3 Departments of Surgery,; 4 Pathology, and; 5 Neurobiology, Duke Medical Center, Durham, NC, U.S.A.; 6 Preston Robert Tisch Brain Tumor Center, Duke Medical Center, Durham, NC, U.S.A.; 7 Neurosurgery Section, Durham VA Medical Center, Durham, NC, U.S.A.

**Keywords:** glioblastoma multiforme, GBM, oncogenomics, genetics, signaling pathways

## Abstract

In the adult population, glioblastoma multiforme is one of the most common primary brain tumors encountered. Unfortunately, this highly malignant tumor represents over 50% of all types of primary central nervous system gliomas. The vast majority of GBMs develops quite rapidly without clinical, radiological, or morphologic evidence of a less malignant precursor lesion (primary or *de novo* GBMs), as compared to secondary GBMs that develop slowly by progression from diffuse low-grade astrocytomas. These GBM subtypes must be kept in mind because they may constitute distinct disease entities. Even though they look histologically quite similar, they likely involve different genetic alterations and signaling pathways. Decades of surgical therapy, radiotherapy, and chemotherapy have failed to drastically change survival. Clearly, we do not fully understand this tumor; however, the exciting genetic revolution in glioma research over the past decade is providing a promising outlook for exploring this tumor at the genetic level. Science has begun to elucidate the numerous genetic alterations and critical signaling pathways, and it has opened new exciting areas of research such as glioma stem cell biology and neoangiogenesis. This work has already begun to improve our understanding of GBM cell proliferation, migration, and invasion. Indeed, exciting novel targeted therapies are making their way to clinical trials based on this increased knowledge. This review provides the current understanding of GBM oncogenomics, signaling pathways, and glioma stem cell biology and discusses the potential new therapeutic targets on the horizon.

## Introduction

In the adult population, glioblastoma multiforme (GBM), is a common and one of the most malignant primary brain tumors, representing up to 50% of all primary brain gliomas.^1^ The majority of GBMs develops rapidly without clinical, radiological, or morphologic evidence of a less malignant precursor tumor (primary or *de novo* GBMs) (Table 1). Secondary GBMs develop slowly by progression from low-grade or anaplastic astrocytomas. Even though they look similar histologically, primary and secondary GBMs appear to constitute distinct disease entities—they affect different age groups, clinically progress at different rates, and involve different genetic alterations and signaling pathways (Table 1).^2^,^3^

**Figure 1.**
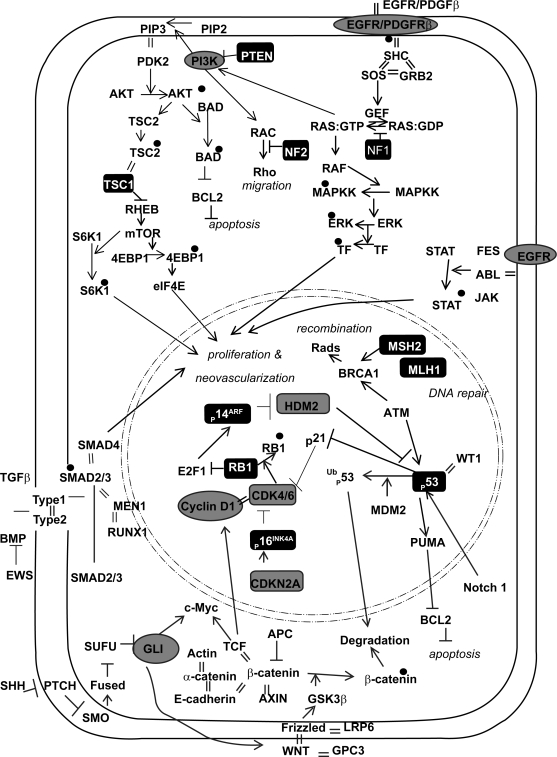
Signaling pathways implicated in GBM. Oncogenes are in gray circles. Tumor suppressor genes are in black boxes. Black dots are phosphate groups. Equal signs represent protein-protein interactions.

Decades of surgical therapy, radiotherapy, and chemotherapy have failed to drastically change survival for GBM. In a recent meta-analysis of 12 randomized clinical trials, the overall survival rate of patients with malignant gliomas was 40% at one year and only slightly higher (46%) after the addition of adjuvant therapies.^4^ We do not have a sufficient understanding of this tumor’s biology; however, the recent genetic revolution in glioma research is providing a promising outlook for exploring this tumor at a more in-depth level. Science has now begun to elucidate a protean network of genetic alterations and critical signaling pathways responsible for GBM initiation, migration, and invasion, opening the door for novel, molecular-based, targeted therapies (Fig. 1). Recent exciting work has introduced the potential for therapeutic targeting of glioma stem cells and tumor neoangiogenic pathways. We now realize that many key pathways are in play. Combination therapies directed at multiple sites have now become common in clinical trials. GBM research is making significant strides forward and exciting novel targeted therapies are making their way to clinical trials. There is real hope that the near future will bring improved survivals to patients afflicted with this universally fatal disease.

## Oncogenomics and Other Genetic Events

### Oncogenomics

Comprehensive genetic screens of GBM have shown that genetic loss is scattered across the entire genome, affecting nearly all chromosomes at frequencies ranging from 2% to 80%. Particularly common regions of loss include areas on 1p, 6q, 9p, 10p, 10q, 13q, 14q, 15q, 17p, 18q, 19q, 22q, and Y.^5^–^10^ Loss of heterozygosity (LOH) on chromosome 10 is the most frequent genetic alteration in GBM, occurring in 60%–80% of cases.^11^ Many GBMs appear to have lost an entire copy of chromosome 10, but several LOH studies have suggested at least three distinct loci to be deleted (e.g., 10p14–p15, 10q23–24, distal to 10q25). This strongly suggests the presence of tumor suppressor genes (TSGs) at these loci.^11^–^13^ Most primary GBMs show loss of the entire chromosome, whereas secondary GBMs with LOH show partial loss of 10q.^14^ Allelic losses on 1p and 7q have also been seen in GBM, but at lower frequencies. Loss of 1p occurs in up to 31% of GBMs^8^,^15^,^16^ and in combination with 19q loss, may indicate a better prognosis and response to therapy.^16^–^18^ Loss of 7q is seen in 9% to 12% of GBMs.^8^,^15^ Loss of gene dosage from numerous genomic alterations has been documented in GBM, such as entire chromosomal loss, partial chromosomal loss, specific allelic loss, inactivating mutations, and promoter methylation. To date, few specific TSGs have been definitively implicated in GBM. It is assumed that many of these losses represent loss of specific TSGs with direct effects on gliomagenesis; however, some of these losses likely represent the inherent genomic instability that develops in tumor cells.

Gains of gene expression due to genetic alterations at the genomic level have also been demonstrated in GBM in the form of duplication of entire chromosomes, intra-chromosomal amplification of specific alleles, extra-chromosomal amplification (often in the form of double minutes [dmins]), and activating mutations.^10^,^19^,^20^ These forms of increased gene expression (oncogenic) occur much less frequently than losses of gene expression as noted above. Clearly, the most common oncogenic event is amplification of the epidermal growth factor receptor (*EGFR)* gene on chromosome 7, often in the form of dmins.^20^,^21^ A few other genes have been clearly implicated as oncogenes (Table 2 and Fig. 1). This collection of oncogenomic events is daunting and difficult to decipher. Recently developed methods for performing genome-wide screens will greatly help to better define these events with respect to tumor initiation, progression, migration, invasion, and survival in preclinical models.

Many genetic alterations, such as gene amplification or deletion, represent direct glioma-inducing events, whereas others indirectly affect gliomagenesis through processes such as DNA instability. The overall mutation rate in somatic human cells is about 1.4 × 10^10^ nucleotides per cell division, translating into about one mutant gene per cell during an individual’s lifespan. The low spontaneous mutation rate in normal cells cannot account for the large mutation rate in GBM. In addition to many of the genetic events noted above, gliomagenesis likely involves errors in DNA replication, DNA repair, chromosomal segregation, and alteration of numerous signaling cascades not directly attributed to genomic mutations. This collection of genetic and cellular alterations gives rise to a tumor cell phenotype, previously described as a “mutator phenotype.”^23^ Central to this mutator phenotype are DNA repair mechanisms. There are at least four major DNA repair pathways that may go awry in GBMs, including nucleotide excision repair, base excision repair, mismatch repair, and direct reversal of lesions in recombination.^23^,^24^ As one example, elevated levels of the DNA repair enzyme O_6_-methylguanine-DNA methyltransferase (MGMT) have been demonstrated in GBM.^25^ This would confer resistance towards alkylating agents. Its expression also correlates with temozolomide (TMZ) treated GBMs.^26^ MGMT specifically removes promutagenic alkyl groups from the O_6_ position of guanine in DNA. Therefore, MGMT protects cells against carcinogenesis induced by alkylating agents. Repair of O_6_-alkylguanine adducts by tumor cells has been implicated in drug resistance, since it reduces the cytotoxicity of alkylating chemotherapeutic agents.^27^ Loss of *MGMT* expression may be caused by methylation of promoter CpG islands, which has been observed in gliomas.^28^,^29^ *MGMT* promoter methylation has been detected in 75% of secondary GBMs, much more than in primary GBMs (36%).^29^ Interestingly, the majority of low-grade gliomas with *MGMT* methylation (92%) contain a mutation in the tumor suppressor gene *p53*, whereas only 39% of those without *MGMT* methylation carry a *p53* mutation.^29^ Furthermore, G:C to A:T transition mutations at CpG sites are significantly more frequent in low-grade gliomas with *MGMT* methylation (58%) than in those without (11%).^29^ This association with increased frequency of *p53* mutations suggests that loss of *MGMT* expression due to promoter methylation frequently occurs at an early stage in the pathway leading to secondary GBMs.

Despite the development of a mutator phenotype and the plethora of genetic alterations it likely entails, there are a discreet number of genetic events and signaling pathways that appear to be central to GBM initiation, migration, invasion, and survival (Fig. 1). Growth factor pathways, such as epidermal and vascular epithelial growth factor receptors (EGFR, VEGFR) play a significant role glioma cell proliferation, migration, and neovascularization. Ras plays a critical intracellular crossroad between numerous pathways, allowing Ras to influence most tumor cell behaviors. P53 and RB1 pathways are often intimately connected in oncology. They both play pivotal roles in regulating cell cycling in response to stimuli for cellular repair or cellular growth. Perhaps due to its homology to proteins that interact with plasma membrane adhesions and the cytoskeletal network, PTEN-related signaling is often implicated in cellular migration in addition to its connection to the Ras cascade.

## Signaling Pathways

### Growth factor tyrosine kinase receptor pathway

In GBM, aberrant EGFR and other tyrosine kinase receptor autocrine signaling pathways may be the most often cited pathways. These lead to robust alterations in cellular development, proliferation, migration, and tumor-induced neovascularization. Growth factor signaling mediated by ligands like EGF activates an intricately complex network of intracellular cascades modulated by G-protein-coupled receptors and second messengers which converge at multiple sites, one of which is Ras. These clearly play a central role in brain gliomagenesis. *EGFR* amplification and overexpression occur in 40%–60% of primary GBMs, and rarely in secondary GBMs.^2^,^21^,^30^,^31^ Amplification of the *EGFR* gene is often associated with structural alterations in the gene. Seven major mutated variants of *EGFR* have been identified, the most common being variant III (*EGFRvIII*), also called *de2–7EGFR* or ρ*EGFR* which is present in 20%–50% of GBMs with *EGFR* amplification.^32^–^35^ *EGFRvIII* results from a nonrandom 801-bp inframe deletion of exons 2–7 of the *EGFR* gene that occurs at the genomic level leading to expression of aberrant transcripts and proteins.^34^ This mutated protein lacks a portion of the extracellular ligand-binding domain as a result of genomic deletions, resulting in a constitutively autophosphorylated receptor, albeit at a lower level than wild type.^36^ In addition to enhancing growth, proliferation, migration, and tumor neovascularization, this truncated receptor also confers resistance to chemotherapies such as cisplatin through modulation of Bcl-XL and caspases in cell death pathways.^37^ Multiple murine glioma models have confirmed the importance of this aberrant growth factor signaling in gliomagenesis.^38^–^40^ Platelet derived growth factor receptor (PDGFR) is a similar receptor expressed in most types of gliomas,^41^ while EGFR is expressed mainly in GBM.^42^ PDGFR signals through phosphoinositide 3-kinase (PI3K) and phospholipase C gamma (PLC-γ).^43^

### Ras signaling

In sporadic GBMs, specific mutations affecting *Ras* have not been detected; however, high levels of Ras guanosine triphosphate (GTP) have been documented in cell lines and primary tumors, suggesting that this signaling pathway is activated by upstream factors such as receptor tyrosine kinase activation (e.g. EGFR or PDGFR).^44^ Another major way of activating this pathway is via the loss of neurofibromin function, the protein product of the large neurofibromatosis 1 (*NF1*) gene. *NF1* has been considered a mutational hot spot in the human genome.^38^ Germline mutations and loss-of-function mutations have been seen in the disease, which includes optic nerve gliomas, astrocytomas, and GBMs.^45^–^47^ *NF1* negatively regulates Ras as an exchange factor converting Ras-GTP to Ras-GDP by its GTPase-activating (Ras-GAP) domain.^38^ Ras-GTP is downstream of growth factor receptors at a major signal transduction crossroad, translating extrinsic messages into the Raf-MAPKK-ERK pathway or into either the PI3K-PKB or the PI3K-Rac-Rho pathway. These influence cell survival and migration. The PI3K-Rac-Rho pathway is involved in cell motility and is negatively regulated by merlin, the protein product of the neurofibromatosis type II (*NF2*) tumor suppressor gene that links the cytoskeleton to the membrane.^48^

In mouse models of GBM, neither activated Ras nor Akt alone induce tumors; however, the combination of both activations can.^40^ Similarly, mouse models deficient in *NF1* and *p53* develop GBM-like tumors with all the characteristic features, including invasion, neovascularity, necrosis, and atypical astrocytes.^49^

### TGF-β signaling

Overexpression or altered signaling of growth factors and their respective pathways is a common theme in GBM. EGFR, VEGF, PDGR, and TGF have all been implicated in GBM. In response to TGF-β stimulation, two TβRI receptor-associated Smads, Smad2 and Smad3, become phosphorylated and activated. After activation, Smads form a heterodimeric complex with Smad4 and translocate to the nucleus to participate in activation of numerous target genes that contribute to proliferation and neovascularization.^50^ TGF-β appears to have alternative mechanisms for promoting tumorigenesis, either as a tumor suppressor gene, a mitogen, or an invasion-promoter. TGF-β can function as a tumor suppressor gene in GBM by inhibiting expression of cdks or downregulating cdk activity by inducing cdk inhibitors p15, p27, and Cip/WAF1/p21.^51^,^52^ Perhaps more interesting is that it can downregulate cell adhesion proteins, induce an epithelial to mesenchymal transition, and thereby, enhance cell migration and invasion.^53^–^55^ TGF-β can alter collagen synthesis, integran expression, cell adhesion to reconstituted basement membrane, and invasiveness in gliomas.^56^,^57^ TGF-β1 and TβRII are expressed in GBM and not in normal brain or low-grade gliomas,^58^ where expression levels are indirectly correlated with survival. TGF-β induces expression of PDGF-A, which may serve as the primary mediator of TGF-β growth stimulatory effects.^59^ Instead of the standard Smad pathway that results in growth inhibition, TGF-β can deviate upstream of Smad to activate other mitogenic pathways implicated in GBM, including MAPK (Ras-Erk) and SAPK (Rho-JNK, TAK1-p38 kinase). These pathways result in activation of different target genes leading to proliferation and transformation. Compared to the other commonly studied signaling pathways in GBM, TGF-β is a unique therapeutic target in that it may represent an important crosslink to various intracellular processes. Unfortunately, its complex and sometimes opposing actions have made it difficult to clearly understand when and how to target it.

### p53-MDM2-p14^ARF^ pathway

The majority of malignant brain tumors, including GBM, demonstrate inactivating mutations in either the p53 and/or retinoblastoma (RB) pathways. 60–63 These two pathways affect numerous cellular functions, but they are most intensely implicated in cell cycling regulation during times of cell repair or cell growth. They interact with each other via p21. P53 is a short-lived transcription factor which is upregulated in response to cellular stress such as radiation exposure, DNA strand breaks, and toxins. It facilitates DNA repair by halting the cell cycle for repair enzymes to work, or if the damage is too great, it induces cell death. As an “apoptostat” protein, it sets a cell’s apoptotic threshold in response to specific endogenous and exogenous insults. Following DNA damage, p53 is activated and induces transcription of genes such as *p21^Waf1/Cip1^*.^64^,^65^ P53 is stabilized by binding to p14^ARF^ and degraded by MDM2.^65^ Secondary GBMs have a higher incidence of p53 mutations (>65%), the majority of which are present in prior lower grade biopsies. 2,31 Amplification of *MDM2* is present in up to 10% of GBMs, and these all appear to be in primary GBMs that lack *p53* mutations.^66^ Loss of p14^ARF^ expression has often been seen in GBMs (76%), and this correlates with homozygous deletion or promoter methylation of the *p14^ARF^* gene.^67^ There is no difference in the overall frequency of *p14^ARF^* alterations between primary and secondary GBMs, but *p14^ARF^* promoter methylation is more frequent in secondary GBMs.^67^ Analysis of multiple biopsies from the same patient reveals that *p14^ARF^* methylation is present in up to a third of lower grade astrocytomas, suggesting an early event in secondary GBMs.^67^

The type and distribution of *p53* mutations may differ between GBM subtypes. In secondary GBMs, 57% of mutations are located in two hotspot codons 248 and 273, whereas in primary GBMs, mutations are more equally distributed through all exons.^2^ Additionally, G:C to A:T mutations at CpG sites, especially in codons 248 and 273, appear to be an early event associated with malignant transformation in the pathway to secondary GBMs. This discrepancy between GBM subtypes is unclear. The less specific pattern of *p53* mutations may simply represent increased genomic instability.

### RB1-p16^INK4a^ pathway

RB1 controls the transition from G1 into S phase of the cell cycle by inhibiting the action of elongation factor E2F1. The cyclin-dependent kinase 4 (CDK4)/cyclin D1 complex phosphorylates the RB1 protein, thereby increasing release of the E2F1 transcription factor that activates genes involved in the G1 to S transition.^68^ p16^INK4a^ binds to CDK4, inhibits the CDK4/cyclin D1 complex, and thus inhibits the G1 to S transition.^68^ Inactivating mutations in *RB1* or the upstream factor *p16^INK4a^* (also called inhibitor of CDK4a), or activating mutations in the downstream factors *CDK4* or *cyclin D*, cause dysregulated control of E2F1. This leads to the expression of S-phase-related genes and uncontrolled cell cycling. Additionally, it leads to expression of anti-apoptotic genes like *Bcl-2*, causing uncontrolled cell proliferation.^69^

The genetic locus *INK4a/ARF* on chromosome 9p21 produces both *p14^ARF^* and *p16^INK4a^* by alternative splicing.^64^ Since p16^INK4a^ negatively regulates CDK4 and p14^ARF^ (p19^ARF^ in mice) inhibits MDM2, blocking rapid ubiquitin-mediated decay of p53, simultaneous inactivation of both genes by a homozygous deletion dysregulates both the RB1 pathway and the p53 pathway. In other words, this single locus drives gliomagenesis. Up to 50% of malignant human gliomas have homozygous deletions that span the reading frame of both genes.^63^ This is one of the most frequent genetic changes in GBM that is acquired during tumor progression. Most GBMs that retain an intact *INK4a/ARF* locus display mutations in other genes of the p53 and the RB pathways, leading to unchecked cell cycling and apoptotic resistance. As alluded to above, p16^INK4a^ is an inhibitor of CDK4, blocks CDK4-dependent phosphorylation of the RB protein^70^ and acts as a negative regulator of cell proliferation. Different mechanisms have been reported for inactivation of the *p16^INK4a^* gene in GBM, including homozygous deletion and hypermethylation of the promoter region.^71^–^74^ In GBM, frequent promoter hypermethylation has been noted for *p14^ARF^* and *RB1*.^75^–^77^

Homozygous *p16^INK4a^* deletions are more frequent in primary than in secondary GBMs.^67^,^78^ However, there is no major difference in overall frequency of any *p16^INK4a^* alteration (including homozygous deletion and promoter methylation) between these GBM subtypes.^67^ Promoter methylation of the *RB1* gene is more frequent in secondary (43%) than in primary GBMs (14%).^79^ There is a correlation between loss of RB1 expression and promoter methylation of the *RB1* gene in GBMs.^79^ *RB1* promoter methylation is not detected in low grade and anaplastic astrocytomas, suggesting that this is a late event in astrocytoma progression.^79^

### PTEN/Akt-1 pathway

Mutations of the TSG *phosphatase tensin homology* (*PTEN*) on chromosome 10q23, also called *MMAC1* and *TEP1*, occur frequently in familial developmental and cancer syndromes such as Cowden-Bannayan syndrome and Lhermitte-Duclos disease.^80^,^81^ Many of these syndromes include GBM as part of the clinical spectrum. PTEN contains a central catalytic phosphatase core domain that negatively regulates PI3K by dephosphorylating phosphatidylinositol-3,4,5 triphosphate (PIP3) and phosphatidylinositol-3,4 diphosphate (PIP2).^82^ The N-terminus of PTEN is homologous to the cytoplasmic proteins tensin and auxilin, which interact with actin filaments at focal adhesions and clathrin-coated vesicles. In the case of mutant PTEN, the elevated lipid second messenger PIP3 is used by PI3K to hyperphosphorylate Akt (also known as protein kinase B [PKB]).^83^ This modulates the activity of proteins that play a critical role in cell survival, invasion, and proliferation.^84^ The catalytic activity toward phosphoinositide substrates is required for growth suppression, and PTEN-mediated growth inhibition is due to G1 cell cycle block rather than induction of apoptosis.^85^ The PTEN C2 domain binds phospholipid membranes and mutations in this domain reduce PTEN’s membrane affinity and ability to suppress growth and motility of GBM cells. Alternatively, tumors with an activated PTEN/Akt pathway may be sensitive to mTOR inhibitors, such as rapamycin.^86^

In GBM, deletions distal to 10q25 (distal to *PTEN*) cover *DMBT1* and *FGFR2* loci,^87^ which suggest that acquisition of the GBM phenotype is associated with loss of other putative TSGs. The *PTEN* gene at 10q23 is mutated in 5%–40% of GBMs.^68^,^80^,^86^,^88^,^89^ *PTEN* mutations are almost exclusively seen in primary GBMs, but rarely in secondary GBMs.^2^ *PTEN* homozygous deletion is rare (<2%).^90^ Promoter methylation may be an alternative mechanism of loss of *PTEN* expression, but the significance of *PTEN* methylation in the evolution of GBMs remains to be determined.^91^ Nonsense mutations (12%) and deletions or insertions leading to stop codons (32%) appear to be more equally distributed throughout the exons, whereas one-third are missense mutations leading to amino acid changes, preferentially located in exons 1–6, regions homologous to tensin, auxilin, and phosphatases.^2^,^92^

### Glioma stem cells

The concept of a cancer stem cell is not a recent phenomenon, but has only recently been extended to brain tumors and exploited as worthy of study for therapy. The well-characterized lineage-specific cell surface markers that define the complex hierarchical model of the hematopoietic system, including hematopoietic stem cells, provided the clues for the first elegant studies identifying cancer stem cells in acute myeloid leukemia^93^ and more recently in gliomas.^94^–^97^ Glioma stem cells (GSCs) represent a small fraction of the tumor cell population that are capable of asymmetric cell division into self-renewing GSCs and differentiating daughter cells that can acquire different phenotypes, subsequently losing their multipotent ability. Their capacity for limitless self-renewal and their ability to repopulate and maintain a heterogeneous tumor indicate that GSCs are a promising target for curative therapies. However, significant fundamental questions still remain about these unique GSCs. A cell capable of initiating a glioma may not necessarily be the same cell responsible for long-term glioma self-renewal; therefore, there may exist subpopulations of “GSC initiators” and “GSC propagators.” The cell of origin for GSCs is still debated with evidence supporting different theories.^98^ These cells may arise from developmentally arrested neural progenitors or from dedifferentiated astrocytes. Heterogeneity within a glioma may not necessarily reflect a stem cell origin of the glioma producing different phenotypes, but could be due to mutations inducing self-renewal properties in progenitor cells with more limited differentiation potential. Clearly, targeting GSCs with therapies poses new dilemmas. Theoretically, a GSC would normally be quiescent, entering the cell cycle only in response to external cues such as growth factors; therefore, quiescent GSCs would unlikely be vulnerable to classic treatments that preferentially target rapidly dividing cells. GSCs represent fundamentally unique glioma cells and as discussed below, they likely depend on genomic alterations and intracellular signaling pathways that differ from non-GSC tumor cells. Therefore, these GSC may contribute to resistance to current therapies as well as future therapies that target genes and proteins that characterize the bulk of the tumor mass. GSCs may also express high levels of drug export proteins, contributing to therapeutic resistance.

Despite the challenges, targeting GSCs is particularly attractive because of the universal pattern of recurrence in these tumors. Neurosurgeons can often remove over 90% of the visible tumor, but the cells that have migrated away from the visible tumor focus will proliferate and lead to the demise of the patient. There is circumstantial evidence that GSC may be related to these migrating glioma cells, so it is reasonable to assume that targeting GSCs will either control migrating cells from the primary focus or limit tumor growth at the secondary focus. Seen throughout all of oncology are the common genetic events and signaling pathways used in cancer cells as well as normal stem cells. Interestingly, neural stem cells have the same tremendous capacity for migration as glioma cells, supporting the idea that some of the migrating glioma cells have GSC properties. If GSCs are found to arise from neural stem cells, then targeting GSC may indeed target the migrating tumor cells and lead to a cure for patients who can tolerate the successful treatment of their primary glioma focus. EGFR overexpression is sufficient to confer migratory potential to neural progenitors supporting the theory that gliomas may originate in progenitors from the subventricular zone (SVZ).^99^ These cells may rapidly migrate to more favorable areas for tumor proliferation, e.g. factors that support better neoangiogenesis.

Targeting GSC will likely have to rely on genomic or proteomic characteristics that differ from other glioma tumor cells. GSCs express immature antigens specific for neural stem and progenitor cells, including the neurofilament protein nestin, the glycoprotein CD133 (a.k.a. prominin-1), Musashi-1 and BMI-1.^94^–^96^ These markers suggest the activation of similar developmentally regulated pathways.^100^ CD133 has been the most prevalent marker for isolating GSCs from GBM.^97^,^101^ CD133+ cells comprise 5%–30% of the tumor cell population, and as few as 100 cells can reproduce tumors in animal models.^101^ A marker of multipotent stem cells in blood and other tissues, much work remains to elucidate the role of CD133 in GSCs. Sonic hedgehog (SHH) and Notch (Fig. 1) are key regulators of neural progenitors in development and have also been found to be altered or overexpressed in GSCs.^102^ SHH has been best studied in the malignant brain tumor, medulloblastoma. However, some evidence supports its role in GBM GSCs as well.^103^ SHH is a critical mitogen for medulloblastoma precursor cells. Hereditary loss of function mutations in the SHH receptor Patched (PTCH) lead to constitutive activation of the SHH pathway and predisposition to medulloblastoma in Gorlin syndrome. The SHH pathway is intimately connected to cell cycling since it inactivates RB1, facilitating the over-expression of cell cycle regulators such as N-myc. Notch activation induces expression of downstream target genes, such as p53, and promotes neural stem cell growth.^104^ It also is better studied in medulloblastoma where activating mutations lead to stem-like cells, but it has been implicated in GSCs as well. Clearly implicated in gliomagenesis, EGFR expression also plays a role in neural progenitor development and is speculated to contribute directly to GSC maintenance.

Olig2 is expressed in myelinating oligodendroglia, but is also in mitogen-treated “transit-amplifying cells” of the SVZ, the presumed site of most neural progenitor cells and a theorized site for the origin of gliomas.^105^ Olig2 and Olig1 are basic-helix-loop-helix transcription factors expressed in neural and oligodendrocyte precursors. Olig2 is almost universally found in NG2-positive glia and required for development of these cells.^106^ NG2 is a Chondroitin sulfate proteoglycan that is thought to be another marker of oligodendrocyte progenitor cells. Olig2 sustains a replication-competent state of neural progenitors^107^ via suppression of p21.^108^ There is much work to be done, but Olig2 and NG2 may be important markers and/or targets for oligodendroglioma stem cells.

There is some interaction between genes known to regulate stem cell proliferation and genetic lesions in malignant gliomas. Some of the most common lesions in malignant gliomas are loss-of-function mutations in p16^Ink4a^ and p19^ARF^ negative regulators of the RB signaling pathway. Gain-of-function mutations in CDK4 are seen, in GBM, activating the RB pathway.^109^ Bmi1, a promoter of neural stem cell self-renewal and neural development, is expressed in most gliomas and promotes malignancy in p16^Ink4a^/p19^ARF^ double-null murine gliomas. 110 As already described, Olig2, a marker of oligodendroglia precursors, is expressed in 100% adult gliomas^111^ and 100% of CD133 + glioma stem cells. Olig2 is required for tumor formation in p16^Ink4a^/p19^ARF^ double null murine gliomas. Recent studies have confirmed the extensively studied role of members of the TGF-β superfamily in morphogenesis and specifically brain development.^112^ As described above, TGF-β may play a significant role in GBM development as well.

Studies such as these strongly support the progenitor cell of origin theory for GSCs. It appears that there may be common genetic gatekeepers, such as Olig2 and SHH, for neurodevelopment and glioma formation. Developing targeted therapies may be developed to kill GSCs, but may also require sophisticated engineering in order to avoid harming normal stem cells. GSC may provide the clues to resistance of this deadly tumor to current therapies. Despite being sensitive to TMZ, the most common chemotherapy in GBM, CD133 + GSCs appear to be quite resistance to radiation therapy.^113^,^114^

### Summary and future hope

Perhaps the greatest mystery in understanding the oncogenomics of GBM is deciding how and when these genetic alterations and signaling cascades interact. Even though only a few specific pathways are consistently highlighted, there are undoubtedly complex interactions among them as well as with additional unknown players. Clearly, many if not most of these genetic events described do not occur in isolation. For example, LOH 10q is not only the most frequent genetic alteration, but typically occurs in the context of other genetic alterations.^2^ This suggests that LOH of 10q plus other genetic events create a genomic environment that collectively contributes to the development of a majority of GBMs. Mutations of *p53*, *p16^INK4a^* deletion, *EGFR* amplification, and *PTEN* mutations show inverse associations with each other, except for a positive correlation between *p16^INK4a^* deletion and *EGFR* amplification.^2^,^115^,^116^ Once a thorough road map for GBM oncogenomics is designed, it should aid in the development of more rational and more effective targeted therapies. Indeed, it is highly plausible that a cocktail of therapies targeting the major intersections in the roadmap for each individual GBM will be required to effectively slow or halt this tumor.

The lack of significant progress in GBM therapy is likely multifactorial, including a lack of oncogenomic understanding, tremendous multi-drug resistance, radioresistance, insufficient preclinical models, and a tenacious blood-brain barrier. Gene expression profiles and preclinical studies have elucidated many of the oncogenomic changes, but basic questions such as the GSC origin and the hierarchy of the oncogenomic events still remain elusive.

The current standard of care for GBM patients begins with gross total resection if possible, followed by concomitant radiotherapy (typically 60 Gy in 2-Gy fractions) and TMZ chemotherapy (75 mg/m^2^ for 42 days). This adjuvant therapy is repeated in a 5/28-day schedule for 5 cycles (150 mg/m^2^) if tolerated. Surgery and radiotherapy have been mainstays in GBM treatment for decades with little modification. TMZ, an alkylating agent, was only recently established in a randomized clinical trial as the standard of care chemotherapy, demonstrating a slight increase in overall survival from 12 to 15 months.^117^

Our current exploration of aberrant molecular and cytogenetic pathways involved in GBM has led to numerous clinical trials testing more specific, molecular-based, therapies for this tumor. EGFR has been an attractive target, especially since it can trigger several downstream signaling pathways (PI3K/AKT/mTOR, Ras/Raf/MAPK, and PKC) and is abnormally activated in some manner in up to 60% of GBMs and 70% of all solid cancers. Targeting strategies have included monoclonal antibodies, bispecific antibodies, toxin-linked conjugates, vaccines, and small-molecule tyrosine kinase inhibitors (TKIs). Phase II clinical trials with EGFR TKIs (ZD-1839, gefitinib; OSI-774, erlotinib) demonstrated some responses, but overall minimal improvement over historical controls in unselected patients.^118^,^119^ Because of the activation of mTOR due to the frequent loss of PTEN in GBM, mTOR inhibitors have also become exciting tools. The mTOR inhibitor CCI-779 (ester of the immunosuppressive agent sirolimus [rapamycin]) did not show efficacy as monotherapy, but may be applicable to combination therapies.^120^ CCI-779 binds FKBP-12 to form a complex that inhibits mTOR, resulting in cell-cycle arrest. The matrix metalloproteinase inhibitor marimastat has been combined with TMZ and demonstrated increased progression-free survival in GBM patients.

These examples of molecular-based targeted therapies being tested in clinical trials represent a new era in GBM therapeutics that bring hope to those afflicted with this fatal disease. With this enthusiasm for testing therapies based on specific GBM biology, we are entering an exciting era in dealing with this deadly tumor. Numerous other targeted therapies have gone through preclinical testing.^121^ Unfortunately, we are still awaiting any major breakthroughs in clinical trials. Further unraveling of the critical genetic alterations that lead to the initiation and progression of GBM may be necessary for future therapies to work.

## Figures and Tables

**Table 1.**
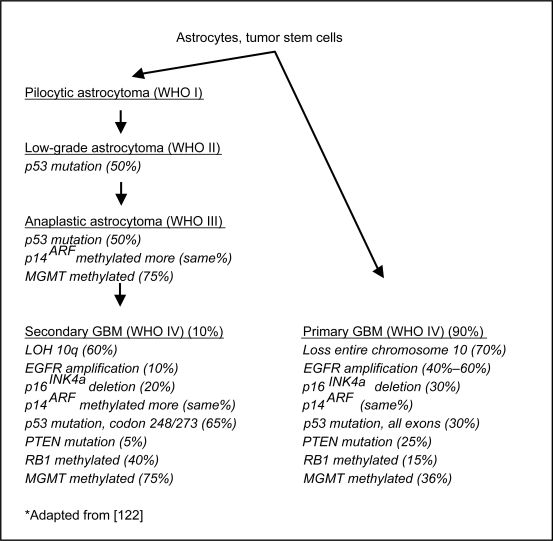
Major genetic alterations in primary and secondary GBM initiation and progression.*

**Table 2.** Genetic alterations in GBM.

**Gene**	**Chromosome**	**Mechanism**	**Frequency**	**Ref.**
Multiple	1p, 6q, 7q, 9p, 10p, 10q, 13q, 14q, 15q, 17p, 18q, 19q, 22q, Y	losses	2%–75%	5–7, 9, 123 8, 10, 15, 16
Multiple	1q, 3q, 4p, 4q, 7p, 7q, 12q, 13q, 19	gains	up to 80%	10, 19, 20
CASP8	2q33	promoter methylation	nd	124
KLF6	10p15	inactivating mutation	12%	125
MGMT	10q26	methylated	up to 75%	28, 29, 126
NF1	17q11	inactivating mutation	nd	38
NF2	22q12	deleted	nd	
p16 and p14	9p21	homozygous deletion	up to 50%	61, 63, 127–129
p16^INK4a^	9p21	deleted, methylated	40%–60%	60, 71–74
p14^ARF^	9p21	deleted, methylated	nd	29
p53	17p13	inactivating mutation	30%–60%	60, 127, 130, 131
PTEN	10q23	inactivating mutation	5%–40%	80, 81, 88, 89, 132
RB1	13q14	inactivating mutation	30%	61, 128, 133
RUNX3	1p36	methylated	nd	134
TES	7q31	methylated	nd	134
TMS1/ASC	16p11	methylated	20%	135
CDK4	12q14	amplified	nd	19, 20
CDK6	7q21	amplified	nd	20
COL4A2	13q34	amplified	nd	20
CSE1L	20q13	amplified	up to 57%	19
EGRF	7p21	amplified (often dmins)	40%–60%	19, 21, 22
EGFRvIII	7p21	amplified	20%–30%	22
ESR	nd	amplified	up to 36%	19
FGR	1p35	amplified	up to 36%	19
GLI	12q13	amplified	nd	19, 136
MDM2	12q15	amplified	<10%	19, 66
MYC	8q24	amplified	nd	19
MYCN	2p24	amplified	nd	19
NRAS	1p13	amplified	nd	19
PDGFRA	4q12	amplified	up to 65%	19, 20
PGY1	7q21	amplified	up to 36%	19
PIK3CA	3q26	activating mutation	nd	136
SLA/LP	4p15	amplified	nd	20
STIM2	4p15	amplified	nd	20
TNFSF13B	13q33	amplified	8%	20
